# Resveratrol's Impact on Vascular Smooth Muscle Cells Hyporeactivity: The Role of Rho-Kinase Inhibition

**DOI:** 10.1155/2020/9012071

**Published:** 2020-01-21

**Authors:** Michał Wiciński, Bartosz Malinowski, Paweł Rajewski, Paweł Szychta, Eryk Wódkiewicz, Maciej Walczak, Karol Górski, Katarzyna Pawlak-Osińska, Maciej Słupski

**Affiliations:** ^1^Department of Pharmacology and Therapeutics, Faculty of Medicine, Collegium Medicum in Bydgoszcz, Nicolaus Copernicus University, M. Curie 9, 85-090 Bydgoszcz, Poland; ^2^The Tadeusz Browicz Provincial Hospital for Infectious Diseases and Observation, 12 św. Floriana St., 85-030 Bydgoszcz, Poland; ^3^Department of Plastic, Reconstructive and Aesthetic Surgery, Faculty of Medicine, Collegium Medicum in Bydgoszcz, Nicolaus Copernicus University, M. Curie 9, 85-090 Bydgoszcz, Poland; ^4^Department of Pathophysiology of Hearing and Balance System, Faculty of Medicine, Collegium Medicum in Bydgoszcz, Nicolaus Copernicus University, M. Curie 9, 85-090 Bydgoszcz, Poland; ^5^Department of Hepatobiliary and General Surgery, Faculty of Medicine, Collegium Medicum in Bydgoszcz, Nicolaus Copernicus University, M. Curie 9, 85-090 Bydgoszcz, Poland

## Abstract

Resveratrol (3,5,4′-trihydroxystilbene) is a chemical compound belonging to the group of polyphenols and flavonoids. The aim of the present study was to determine the influence of resveratrol application along with certain modulating factors, such as 8Br-cGMP-activator of cGMP-dependent protein kinases, HA-1077-Rho-kinase inhibitor, and Bay K8644-calcium channel agonist, on VMSCs constriction triggered by phenylephrine. Resveratrol at a dose of 10 mg/kg/24 h administered for 4 weeks reduced the reactivity of the arteries to the pressure action of catecholamines. Tests performed after four weeks of resveratrol administration showed that 8Br-cGMP at the concentrations of 0.01 mM/l and 0.1 mM/l intensifies this effect. Simultaneous resveratrol and Bay K8644 administration led to a significant decrease in contractility compared to the vessels collected from animals (Res−). This effect was dependent on the concentration of Bay K8644. Resveratrol seems to be counteractive against Bay K8644 by blocking L-type calcium channels. As the concentration of HA-1077 increased, there was a marked hyporeactivity of the vessels to the pressure effects of phenylephrine. The results indicate synergy between resveratrol and Rho-kinase inhibition.

## 1. Introduction

Resveratrol (3,5,4′-trihydroxystilbene) is a chemical compound belonging to the group of polyphenols and flavonoids. They are also referred to as phytoestrogens—the plant hormones. The natural sources of resveratrol are mulberry, rhubarb, peanuts, and eucalyptus, Scots pine, or Japanese knotweed [1]. It is believed, however, that the main source of resveratrol is grapes, in particular, their peel and seeds, in which the resveratrol concentration can be as high as 50–100 *μ*g/g. No negative effects of resveratrol have been found, even at very high doses reaching 1000-fold resveratrol concentration in red wine administered to rats for 28 days [2]. Following oral administration in humans, approximately 70% of resveratrol is absorbed [3]. Resveratrol and its metabolites have an affinity for numerous cytochrome P450 isoenzymes (CYP450). The first studies describing the beneficial action of resveratrol in the prophylaxis of coronary artery disease appeared at the beginning of the 20th century [4].

Vascular smooth muscle cells (VSMCs) are widely considered as the elements of the pathogenesis of hypertension and atherosclerosis [5]. Their contraction mechanism is dependent on the Ca^2+^ influx [6]. The process of Ca^2+^ binding with calmodulin in the VSMCs causes myosin light chain phosphorylation and consequent constriction [7]. In the cell membrane, DAG (diacylglycerol) stimulates the activity of protein kinase C, which is responsible for the inclusion of Ras superfamily members into the signaling pathway [8]. The interaction between one of their representatives—member A (RhoA) and Rho-kinase (ROK)—results in a decrease of myosin light chain phosphatase (MLCP) activity. Thus, inhibited phosphatase is unable to dephosphorylate myosin light chain. This process increases the sensitivity of the contractile apparatus of the VSMCs to Ca^2+^, which maintains the constriction [9]. The aim of the present study was to determine the influence of resveratrol application along with the inhibition of certain molecular factors on VMSCs constriction triggered by phenylephrine.

## 2. Materials and Methods

The study was conducted on 25 male rats of the Wistar strain (mean age of 3 months), weighing 250–350 g (Animal Laboratory Breeding, Brwinów, Poland). To perform a pharmacometric assessment, isolated caudal arteries were used, as a classic model of the resistance artery. Laboratory animals were maintained in a 12-hour light/dark cycle. The room temperature was 20-21°C, and humidity was 50–60%. Animals were provided with food and water ad libitum day and night.

The study was divided into two experimental phases. In the first phase, animals received a drinking water solution of resveratrol per os at a dose of 10 mg/kg/24 h for 4 weeks. The second phase of the pharmacometric studies was performed on isolated caudal arteries. 120 mg/kg of urethane (Honeywell International Inc., Seelze, Germany) was used for anesthesia. Rats were euthanatized by dislocating the cervical vertebrae. The local Bioethics Committee (approval no. 17/2015) has given assent on conducting the tests. The experiment was carried out in accordance with the United States NIH guidelines.

### 2.1. Reagents and Solutions

The following reagents were used in the experiment: resveratrol (Tocris Bioscience, Bristol, UK) (10 mg/kg/24 h), calcium Krebs liquid composed of NaCl (71.8 mM/l), KCl (4.7 mM/l), NaHCO_3_ (28.4 mM/l), CaCl_2_ (1.7 mM/l), MgSO_4_ (2.4 mM/l), KH2PO_4_ (1.2 mM/l), glucose (11.1 mM/l), and 8Br-cGMP (Tocris), an activator of cGMP-dependent protein kinases, HA-1077 (Tocris) Rho-kinase inhibitor, and Bay K8644 (Tocris), a calcium channel agonist.

### 2.2. Preparation of Arteries and Measurements

After the removal of the tissue surrounding the artery, the proximal section without branchlets of 2-3 cm in length was cannulated and placed in a vertical position in a 20 ml isolated organ bath. The cannula was inserted from the proximal side of the artery and then combined with the perfusion system and a set for continuous measurement and recording of the perfusion pressure. The applied resting tension of 0.5 g has been determined experimentally. In the initial phase of the experiment, the organs were stabilized for 2 hours in an oxygenated Krebs liquid (Merck KGaA) at 37.0°C and pH 7.4. The perfusate flow was obtained with a peristaltic pump, gradually increasing it from 0.25 to 1.0 ml/min, until the optimal pressure in the range of 2–4 kPa was reached. The modulating reagents were administered directly to the incubation vessel. Arterial constriction was induced by phenylephrine-agonist of *α*1-adrenergic receptors. The change in the vascular smooth muscle reactivity was analyzed by constant monitoring of perfusion pressure using BPR-1 and BPR-2 transducers (Experimetria Ltd.) connected to the recorder. The intervals between the administration increasing doses of phenylephrine depended on achieving stabilization of the isolated vessel and were different for the tested compounds. We strove to achieve relatively constant, stable pressures before administrating higher phenylephrine concentration. The mean maximal pressures elected by maximal phenylephrine dose were 11.87 ± 1.33 kPa. This pressure was determined as *E*_max_.

### 2.3. Statistical Analysis

The calculations were performed in the STATISTICA12 program. In order to assess the compatibility of distributions with the normal distribution, the Shapiro-Wilk test was used. The ANOVA test was used to compare the values within groups. The graphs show the mean value with the standard deviation or the medians. The level of statistical significance of the applied tests was *p* < 0.05. The concentration-response curves (CRCs) were determined using the method of increasing concentrations of van Rossum with subsequent modifications of the method. An agonist concentration value corresponding to 50% of the maximum effect was defined as EC50 [10–12]. The presented points are mean values ± SD.

The control curve was determined based on the analysis of the reactivity of the smooth muscle of the arteries with endothelium isolated from animals that did not receive a resveratrol (Res−)—control group. The CRC (concentration-response curve) for resveratrol was calculated based on the analysis of smooth muscle reactivity after four weeks of resveratrol administration to the test group (Res+). The points on the graphs represent the mean values for *n* = 24 trials ± SD (number of partial CRC curves used for the calculation).

## 3. Results

**Figure 1:**
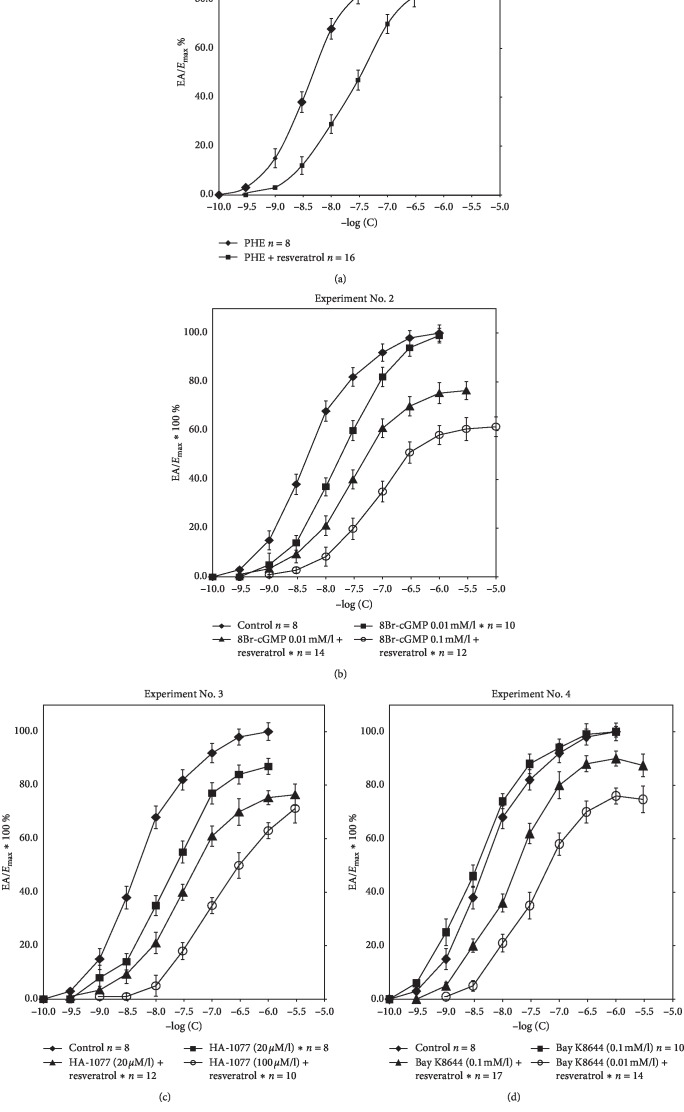
(a) Experiment No. 1. Concentration-response curves (CRCs) determined for control conditions and for resveratrol. ^*∗*^The curves with a maximum effect of 20–80% differ significantly from the control values (*p* < 0.05). (b) Experiment No. 2. Concentration-response curves (CRCs) determined under control conditions for phenylephrine, 8Br-cGMP (0.01 mM/l), and resveratrol with 8Br-cGMP at concentrations of 0.01 mM/l and 0.1 mM/l. ^*∗*^The curves with a maximum effect of 20–80% differ significantly from the control values (*p* < 0.05). (c) Experiment No. 3. Concentration-response curves (CRCs) determined under control conditions for phenylephrine, HA-1077 (20 *μ*M/l), and resveratrol with HA-1077 in concentrations of 20 *μ*M/l and 100 *μ*M/l. ^*∗*^The curves with a maximum effect of 20–80% differ significantly from the control values (*p* < 0.05). (d) Experiment No. 4. CRCs determined under control conditions for phenylephrine, Bay K8644 (0.1 mM/l), and resveratrol with Bay K8644 at concentrations of 0.1 mM/l and 0.01 mM/l.

Resveratrol at a dose of 10 mg/kg/24 h, administered for 4 weeks, reduced the reactivity of the arteries to the pressure action of catecholamines. Experiment No. 1 illustrates the shift of CRC to the right towards higher concentrations for the *α*1-adrenergic agonist phenylephrine (PHE) with the reduction of the maximal effect of *E*_max_ by 18% (*p* < 0.0001) in animals from the study group. The final pressure in Res + group was 7.65 ± 0.79 kPa. The pharmacometric analysis showed a significant increase in EC50 values in the presence of resveratrol (EC50 = 4.53 ± 1.2 × 10^−8^ M/l vs .5.33 ± 1.7 × 10^−7^ M/l, *p* < 0.0001). All data are means ± SD (Figure 1).

8Br-cGMP at a concentration of 0.01 mM/l induces arterial hyporeactivity in both experimental (Res−) and (Res+) groups. In the presence of 8Br-cGMP (Res− group), a parallel shift of CRC to the right was observed without a significant reduction of the maximum effect. Tests performed after four weeks of resveratrol administration showed that 8Br-cGMP at concentrations of 0.01 mM/l and 0.1 mM/l intensifies this effect. In the presence of resveratrol, a significant reduction of the maximum effect, depending on the concentration of 8Br-cGMP, has been demonstrated. *E*_max_ values were 76.4 ± 3.7 and 61.6 ± 4.0 (*p* < 0.0001). Differences were observed only between groups (Res−), in which the EC50 coefficients were 4.53 ± 1.2 × 10^−8^ M/l and 2.77 ± 0.56 × 10^−7^ M/l, respectively (*p* < 0.0001) (Table 1). All data are means ± SD.

CRCs for PHE in the presence of HA-1077 (20 *μ*M/l) were determined on the basis of the analysis of the smooth muscle response of the arterial smooth muscle with the vascular endothelium of rats from the control group (Res−). Curves HA-1077 (20 *μ*M/l) + resveratrol and HA-1077 (100 *μ*M/l) + resveratrol were determined after four weeks of the administration of the compound and the removal of caudal arteries. Rho-kinase inhibitor (ROCK)-HA-1077 at a concentration of 20 *μ*M/l reduces arterial responsiveness to phenylephrine. In the presence of HA-1077 (Res−), a significant CRC shift was observed with the reduction of the maximum effect by 13% ± 3.0% (*p* < 0.0001). Arteries collected from rats receiving resveratrol show similar reactivity. In the presence of HA-1077, at concentrations of 20 *μ*M/l and 100 *μ*M/l, CRCs are significantly shifted towards higher concentrations for phenylephrine with a simultaneous reduction of the maximum effect. The calculated *E*_max_ values in these groups are similar (76.4 ± 4.0% and 70 ± 5.4%); however, at the concentration of HA-1077 (100 *μ*M/l), there is clearly greater hyporeactivity of the arteries to analogous concentrations of phenylephrine. All data are means ± SD.

CRCs for PHE in the presence of Bay K8644 were determined on the basis of the analysis of the reactivity of the vascular smooth muscle of vessels collected from the group (Res−). CRCs Bay K8644 (0.01 mM/l) + resveratrol and Bay K8644 (0.1 mM/l) + resveratrol were determined after resveratrol treatment in the group (Res+). The figure illustrates the changes in vasomotor smooth muscle reactivity in the control group (Res−) and the test group after a four-week administration of resveratrol. Bay K8644 is a direct activator of voltage-dependent calcium channels type L. Bay K8644 at a concentration of 0.1 mM/l did not affect the shape of CRC and % *E*_max_ determined for arteries taken with rat vascular endothelium (Res−). Arteries from rats receiving resveratrol in the presence of Bay K8644 showed a significant reduction of the maximum effect (87.4 ± 4.2% and 74.7 ± 5.0, *p* < 0.0001). This effect was dependent on the concentration of Bay K8644. Vascular reactivity was significantly reduced at the lower concentration of Bay K8644–0.01 mM/l (Table 2). CRCs designated for groups (Res+) were significantly shifted to the right with the reduction of the maximum effect. The EC50 coefficients did not differ significantly within the groups (Res−) and were 4.53 ± 1.2 × 10^−8^ M/l and 1.62 ± 0.43 × 10^−8^ M/l. Incubation of arteries (Res+) with Bay K8644 at a concentration of 0.1 mM/l resulted in a significant increase in EC50 compared to the coefficient value determined for Bay K8644 in the group (Res−)—1.62 ± 0.43 × 10^−8^ M/l vs. 5.17 ± 0.72 × 10^−7^ M/l (*p* < 0.0001). There were no EC50 differences in the (Res+) group depending on the Bay K8644 concentration used. All data are means ± SD.

The mean initial pressures in the control and study groups were 4.15 ± 1.37 kPa and 3.82 ± 0.83 kPa, respectively, while the maximal pressures were 11.87 ± 1.33 kPa and 7.65 ± 0.79 kPa. Tail arteries isolated from animals from the study group (Res+) showed a significant reduction in the final pressure in response to the analogous concentrations of phenylephrine (*p* < 0.0001). sAll data are means ± SD.

Incubation of tail arteries with 8Br-cGMP at a concentration of 0.01 M/L (Res−) results in a statistically significant reduction in maximal pressures (11.87 ± 1.33 kPa vs. 8.63 ± 0.45; *p* < 0.0001). Lower maximal pressures were observed in the group of animals receiving resveratrol in response to PHE-induced contraction in the presence of 8Br-cGMP at both concentrations—0.01 M/L and 0.1 M/L (6.33 ± 0.75 kPa vs. 4.5 ± 0.68; *p* < 0.001). All data are means ± SD.

Arteries collected from rats (Res−) in the presence of HA-1077 at a concentration of 20 *µ*M/l show significantly lower maximal pressures (11.87 ± 1.33 kPa vs. 8.32 ± 0.51 (*p* < 0.0001)). In the group of animals receiving resveratrol, there was a reduction in *p*_k_ in response to the phenylephrine-induced contraction in the presence of HA-1077 at 20 *μ*M/l and 100 *μ*M/l (5.57 ± 0.72 kPa vs. 3.91 ± 0.56 kPa; *p* < 0.001). All data are means ± SD.

The mean maximum perfusion pressure obtained in the control group (Res−) was 11.87 ± 1.33 kPa. Bay K8644 at a concentration of 0.1 mM/l induced a statistically insignificant increase in *p*_*k*_-13.91 ± 1.5 kPa. A significant reduction in *p*_*k*_ was observed in the group of animals receiving resveratrol in the presence of Bay K8644 and this effect was dependent on the concentration of L-type calcium channel activator (10.85 ± 0.86 kPa and 7.69 ± 0.73; *p* < 0.0001). All data are means ± SD.

## 4. Discussion

Our study using classical pharmacometric methods revealed complex mechanisms of vascular smooth muscle hyporeactivity after four weeks of resveratrol administration. The overall assessment of vasoreactivity in the presence of resveratrol should take into account both the mechanisms associated with the receptor pathways and the extrareceptor components, such as G-protein-coupled enzymes, ion channels, or regulation at the level of endothelial cells. According to the research protocol, vasoconstriction was induced by increasing concentrations of phenylephrine. In the presence of the α1 agonist, a significant decrease in vasoconstriction was observed in the group of animals that received resveratrol. These changes are illustrated in Experiment No. 1 and Table 3. The hyporeactivity was confirmed by a significant increase in the EC50 coefficient value in the study group and by lower maximal pressure values. Fernandez-Morales et al. observed similar effects at nanomolar concentrations of resveratrol [13]. Kline and Karpiński's [14] study showed that resveratrol also depends on nonreceptor vasorelaxant mechanisms. In the assessment of the potential impact of cyclic nucleotides in resveratrol-induced hyporeactiveness, 8Br-cGMP, a low molecular weight analogue with high tissue penetration, was used in two concentrations: 0.01 mM/l and 0.1 mM/l (Experiment No. 2, Tables 1 and 4). Significant lower arterial reactivity was observed after four weeks in animals that received resveratrol at a dose of 10 mg/kg/24 h. Also, the maximal pressure values in the (Res+) group decreased significantly with increasing the concentration of 8Br-cGMP. The reduction of vascular resistance caused by cGMP results from the activation of the PKG. The concentration of cGMP (cyclic guanosine monophosphate) is regulated by guanylate cyclase, catalyzing the synthesis of cyclic nucleotide, NO (nitric oxide), and PDE (phosphodiesterase), which induces its hydrolysis. Numerous studies confirm the activation of the cGMP/PKG pathway (protein kinase G) by resveratrol; however, the mechanism of this process has not yet been clarified. Xi et al. [15] have shown that rat cardiomyocytes exposed to resveratrol at a concentration of 10 *μ*M for 10 minutes showed no increase in fluorescence DAF-FM, indicating that the activation of the cGMP/PKG does not occur through the release of NO. Our previous studies on isolated arteries with endothelium have confirmed the statement [16]. The authors postulate that resveratrol inhibits the PDE5 (phosphodiesterase type 5) activity. Thus, the increase in cGMP concentration is considered in the aspect of inhibiting its hydrolysis and not the direct activation of guanylate cyclase [15].

In our study, the effect of resveratrol on Rho-kinase activity was analyzed. To this end, arteries collected from both groups of animals along with endothelium were incubated with a specific inhibitor of the enzyme, HA-1077, using concentrations of 20 *μ*M/l and 100 *μ*M/l, respectively, in series of experiments. As the concentration of HA-1077 increased, there was a marked hyporeactivity of the vessels to the pressure effects of phenylephrine. It was shown that arteries collected from animals that received resveratrol for four weeks showed an attenuation of vasoconstriction compared to the analogous concentration of phenylephrine for the arteries from the control group (Experiment No. 3, Tables 5 and 6) Also, the maximal pressure values in the (Res+) group were significantly lower in reference to the study group. These results indicate synergy between resveratrol and HA-1077 (analysis using the Loewe Additivity model). This confirms the proposed hypothesis that resveratrol may be a potential inhibitor of Rho kinase in smooth muscle. In the literature, we can find data confirming the inhibition of ROCK by resveratrol already at the concentration of 0.03 mM/l. In studies evaluating isometric contraction of isolated aortic rings, it was shown that the mechanism of relaxation underlying the action of resveratrol is associated with the inhibition of Rho kinase and phosphorylation of MYPT1 (a myosin light chain phosphatase component). This effect is not dependent on vascular endothelial function [17]. Studies in mice have also shown that resveratrol reduces the angiotensin II response by activating AMPK and inhibiting RhoA/ROCK [18].

In numerous publications, we find information on the regulation of calcium homeostasis by resveratrol. Due to the relatively good penetration through biological membranes, resveratrol can modulate calcium influx from both intracellular and extracellular fractions [19]. In our research, this process was analyzed in relation to the extracellular pool. The applied L-Bay calcium channel agonist at concentrations of 0.1 mM/l and 0.01 mM/l showed synergistic activity with the *α* 1-adrenergic receptor agonist phenylephrine (Experiment No. 4, Table 7). Bay K8644 increased the influx of Ca^2+^ ions; however, arteries from rats after resveratrol administration showed significantly less contractility compared to the vessels collected from animals (Res−). The EC50 and *E*_max_ pharmacokinetic ratios obtained on the basis of calculations for both groups of animals are presented in Table 2. In their interpretation, attention should be paid to the reduction of the maximum effect in the group of animals (Res+). Vascular hyporeactivity occurring despite the incubation of collected arteries with Bay K8644 indicates a noncompetitive antagonism between reagents. Resveratrol is counteractive against Bay K8644 by blocking L-type calcium channels. These results are corroborated by studies published by Hsia et al., in which resveratrol reduced the contraction of the uterine smooth muscle induced by the activation of L-type calcium channels by Bay K8644. The compound has an analogous activity to 1,4-dihydropyridine derivatives. The complex mechanism of smooth muscle hyporeactivity is demonstrated by the fact that resveratrol reduced its reactivity in the case of induction of PGF contraction (2*α*), oxytocin, acetylcholine, and carbachol. All of these receptors have a similar linkage pathway to GPCRs which ultimately leads to an increase in the concentration of calcium ions in the cell [20]. It is possible that some of the abovementioned mechanisms of resveratrol action are interconnected. Rho kinase seems to play an important role in the mechanism of VSMC constriction. The inhibition of the kinase appears to reduce contractile responsiveness of the arterial muscle layer. Our previous study [21] confirms the theory of the so-called “sensitization” of myosin light chain to Ca^2+^. The term describing this phenomenon was introduced by Somlyo and Somlyo [22, 23] and applies to the RhoA/Rho-kinase signaling pathway that inhibits the dephosphorylation of myosin light chain by the inactivation of MLCP. The researchers proposed a mechanism that is connected with phosphorylation of the M110–130 regulatory chain. Moreover, they pointed out that ROK activity can be inhibited by specific RhoA inhibitors or by acting on GTP/GDP balance [23]. The alteration between active GTP-connected and inactive GDP connected forms of RhoA seems to be dependent on Rho guanine nucleotide exchange factors (Rho-GEFs). The aforementioned MLPC is known to be attenuated by phosphorylated CPI-17, a downstream regulator in Rho-kinase cascade. The downregulation of certain Rho-GEFs (including Arhgef25, Arhgef11, and Arhgef12) and enhanced CPI-17 phosphorylation observed by Behuliak et al. [24] in adult SHR (spontaneously hypertensive rats) might be consistent with the response to Rho-kinase inhibition [21]. To fully elucidate if the changes in the Rho-kinase pathway are in the form of a compensation of increased sympathetic tension and RAS activity, further studies are surely needed. The rise of Ca^2+^ concentration in VSMCs activates RhoA. The specific mechanism has not been fully clarified; however, the model of the interaction between Ca/CaM complex and RhoGEF (guanine nucleotide exchange factor) has been proposed. Furthermore, phosphorylated Rho (Rho-GTP) triggers ROK [25]. In spite of the fact that ROK activity may be activated by Ca^2+^, some researchers believe that RhoA–ROK signaling pathway may itself mediate the influx of the extracellular Ca^2+^ to the cytoplasm [26, 27].

## 5. Conclusions

The reactivity of arterial smooth muscle cells collected with endothelium after four weeks of resveratrol administration is a complex process. Resveratrol seems to be counteractive against Bay K8644 by blocking L-type calcium channels. Increasing concentrations of 8Br-cGMP caused a significant diminishment in the maximal pressure values in the resveratrol treated group. Presented results indicate synergy between resveratrol and Rho-kinase inhibition. Further research is required to confirm the mechanisms observed above.

## Figures and Tables

**Table 1:** Values of EC 50 and *E*_max_ in the control and test groups: resveratrol + 8Br-cGMP 0.01 M/l and 8Br-cGMP 0.1 M/l.

	*n* ^1^	EC_50_ (M/l)	*E*_max_ (%)^2^	*p*
Control (Res−)	8	4.53 ± 1.2 × 10^−8^	100.0	—
8Br-cGMP 0.01 M/l (Res−)	10	2.77 ± 0.56 × 10^−7^^*∗*^*p* < 0.0001	99.0 ± 3.0	ns^a^
8Br-cGMP 0.01 M/l + resveratrol	14	3.43 ± 0.43 × 10^−7^	76.4 ± 3.7	*p* < 0.0001^b^
8Br-cGMP 0.1 M/l + resveratrol	12	3.43 ± 0.43 × 10^−7^	61.6 ± 4.0	*p* < 0.0001^c^

^1^The number of CRC used for the calculation; ^2^*E*_max_ calculated as a % of the maximum tissue response to PHE; ^a^*p* value calculated relative to the control (Res−); ^b^*p* value calculated relative to 8Br-cGMP 0.01 M/l (Res−); ^c^*p* value calculated relative to 8Br-cGMP 0.01 M/l (Res+). All data are means ± SD.

**Table 2:** Values of EC 50 and *E*_max_ in the control and test groups: resveratrol + Bay K8644 in concentrations of 0.1 mM/l and 0.01 mM/l.

	*n* ^1^	EC_50_ (M/l)	*E*_max_ (%)^2^	*p*
Control (Res−)	8	4.53 ± 1.2 × 10^−8^	100.0	—
Bay K8644 (0.1 mM/l) (Res−)	10	1.62 ± 0.43 × 10^−8^	100 ± 2.0	ns^a^
Bay K8644 (0.1 mM/l) + resveratrol	17	5.17 ± 0.72 × 10^−7^^*∗*^^*∗*^*p* < 0.001^a^	87.4 ± 4.2	*p* < 0.0001^b^
Bay K8644 (0.01 mM/l) + resveratrol	14	2.13 ± 0.21 × 10^−7^	74.7 ± 5.0	*p* < 0.0001^c^

^1^The number of CRCs used for the calculation; ^2^*E*_max_ calculated as a % of the maximum tissue response to PHE; ^a^*p* value calculated relative to the control (Res−); ^b^*p* value calculated relative to Bay K8644 (0.1 mM/l) (Res−); ^c^*p* value calculated relative to Bay K8644 (0.1 mM/l) (Res+). All data are means ± SD.

**Table 3:** Mean values of initial pressure (*p*_*o*_) and maximal pressure (*p*_*k*_) induced by phenylephrine (PHE) in calcium fluid in the control (Res−) and test (Res+) groups.

	*n* ^1^	*p*_*o*_ (kPa)	*p*_*k*_ (kPa)
Control (Res−)	8	4,15 ± 1,37	11,87 ± 1,33
Resveratrol (Res+)	16	3,82 ± 0,83	7,65 ± 0,79^*∗*^

^1^Number of CRCs used for pressure calculations; ^*∗*^*p*_*k*_ (Res+) vs. *p*_*k*_ (Res−) *p* < 0.0001.

**Table 4:** Mean values of initial pressure (*p*_*o*_) and maximal pressure (*p*_*k*_) induced by phenylephrine (PHE) in the control group (Res−) and test group: resveratrol + 8Br-cGMP 0.01 M/l and 8Br-cGMP 0.1 M/l.

	*n* ^1^	*p*_*o*_ (kPa)	*p*_*k*_ (kPa)
Control (Res−)	8	4.15 ± 1.37	11.87 ± 1.33
8Br-cGMP 0.01 M/l (Res−)	10	3.28 ± 1.12	8.63 ± 0.45^a^^*∗*^^*∗*^*p* < 0.0001
8Br-cGMP 0.01 M/*l* + resveratrol	14	4.11 ± 0.63	6.33 ± 0.75^b^^*∗*^^*∗*^*p* < 0.0001
8Br-cGMP 0.1 M/*l* + resveratrol	12	2.76 ± 1.92	4.5 ± 0.68^c^^*∗*^^*∗*^*p* < 0.0001

^1^Number of CRCs used for pressure calculations; ^a^*p* value calculated relative to control (Res−); ^b^*p* value relative to 8Br-cGMP 0.01 M/l (Res−); ^c^*p* value relative to 8Br-cGMP 0.01 M/l (Res+).

**Table 5:** Values of EC 50 and *E*_max_ in the control and test groups: resveratrol + HA-1077 in concentrations of 20 *μ*M/l and 100 *μ*M/l.

	*n* ^1^	EC_50_ (M/l)	*E*_max_ (%)^2^	*p*
Control (Res−)	8	4.53 ± 1.2 × 10^−8^	100.0	—
HA-1077 (20 *µ*M/l) (Res−)	8	1.49 ± 0.34 × 10^−7^^*∗*^*p* < 0.0001^a^	87.0 ± 3.0	^*∗*^ *p* < 0.001^a^
HA-1077 (20 *µ*M/l) + resveratrol	12	2.49 ± 0.81 × 10^−7^	76.4 ± 4.0	*p* < 0.0001^b^
HA-1077 (100 *µ*M/l) + resveratrol	10	5.02 ± 0.73 × 10^−7^	70 ± 5.4	ns^c^

^1^The number of CRC used for the calculation; ^2^*E*_max_ calculated as a % of tissue maximal response to PHE; ^a^*p* value calculated value relative to control (Res−); ^b^*p* value calculated against HA-1077 (20 *μ*M/l) (Res−); ^c^*p* value calculated against HA-1077 (20 *μ*M/l) (Res+). The calculated values of the antagonist concentrations corresponding to 50% of the maximum effect are summarized in the table above. In the presence of HA-1077, they were 1.49 ± 0.34 × 10–7 M/l, 2.49 ± 0.81 × 10–7 M/l, and 5.02 ± 0.73 × 10–7 M/l. Significant EC50 differences were observed between the control group (Res−) and HA-1077 (Res−) at a concentration of 20 *μ*M/l-4.53 ± 1.2 × 10–8 M/l. 1.49 ± 0.34 × 10–7 M/l (*p* < 0.0001). All data are means ± SD.

**Table 6:** Mean values of initial pressure (*p*_o_) and maximal pressure (*p*_*k*_) induced by phenylephrine (PHE) in the control group (Res−) and the test group: resveratrol + HA-1077 in concentrations 20 *µ*M/l and 100 *µ*M/l.

	*n* ^1^	*p*_*o*_ (kPa)	*p*_*k*_ (kPa)
Control (Res−)	8	4.15 ± 1.37	11.87 ± 1.33
HA-1077 (20 *µ*M/l) (Res−)	8	3.82 ± 0.83	8.32 ± 0.51^a^^*∗*^^a^*p* < 0.0001
HA-1077 (20 *µ*M/l) + resveratrol	12	2.52 ± 1.05	5.57 ± 0.72^b^^*∗*^^b^*p* < 0.0001
HA-1077 (100 *µ*M/l) + resveratrol	10	1.93 ± 1.24	3.91 ± 0.56^c^^*∗*^^c^*p* < 0.0001

^1^Number of CRCs used for pressure calculations; ^a^*p* value calculated relative to the control (Res−); ^b^*p* value calculated relative to HA-1077 (20 *µ*M/l) (Res−); ^c^*p* value calculated relative to HA-1077 (20 *µ*M/l) (Res+).

**Table 7:** Mean values of initial pressure (*p*_*o*_) and maximal pressure (*p*_*k*_) induced by phenylephrine (PHE) in the control group (Res−) and the test group: resveratrol + Bay K8644 in concentrations 0.1 mM/l and 0.01 mM/l.

	*n* ^1^	*p*_*o*_ (kPa)	*p*_*k*_ (kPa)
Control (Res−)	8	4.15 ± 1.37	11.87 ± 1.33
Bay K8644 (0.1 mM/l) (Res−)	10	4.0 ± 1.1	13.91 ± 1.5
Bay K8644 (0.1 mM/l) + resveratrol	17	3.28 ± 1.24	10.85 ± 0.86^a^^*∗*^^*∗*^*p* < 0.0001
Bay k8644 (0.01 mM/l) + resveratrol	14	2.48 ± 0.82	7.69 ± 0.73^b^^*∗*^^*∗*^*p* < 0.0001

^1^Number of CRCs used for pressure calculations; ^a^*p* value calculated in relation to Bay K8644 (0.1 mM/l) (Res−); ^b^*p* value calculated relative to Bay K8644 (0.1 mM/l) (Res+).

## Data Availability

The data used to support the findings of this study are available from the corresponding author upon request.
